# After 30 Years of Study, the Bacterial SOS Response Still Surprises Us

**DOI:** 10.1371/journal.pbio.0030255

**Published:** 2005-07-12

**Authors:** Bénédicte Michel

## Abstract

The bacterial SOS response kicks in when bacteria experience DNA damage, and helps the organisms correct and survive DNA damage events. This primer provides a foundation for understanding these events.

In order to survive in various environmental conditions, cells have a repertoire of genes that they can choose to express or silence according to their needs. Among this vast collection of genetically controlled networks, the SOS response is an inducible DNA repair system that allows bacteria to survive sudden increases in DNA damage. The importance of the SOS response is underscored by the observation that this regulatory network is widely present in bacteria, reflecting the need for all living cells to maintain the integrity of their genome.

## Jump-Starting DNA Repair

The first experimental support for the existence of an inducible DNA repair network in Escherichia coli was found 30 years ago by Miroslav Radman, who introduced the term “SOS response” to describe this network [1]. Two proteins play key roles in the regulation of the SOS response: a repressor named LexA and an inducer, the RecA filament. During normal growth, the LexA repressor binds to a specific sequence—the SOS box, present in the promoter region of SOS genes—and prevents their expression. SOS genes are repressed to different degrees under normal growth conditions. This depends on the exact sequence of their SOS box (the region of a promoter that is recognized by LexA), its position in the promoter region, and the strength of the promoter.

**Figure 1:**
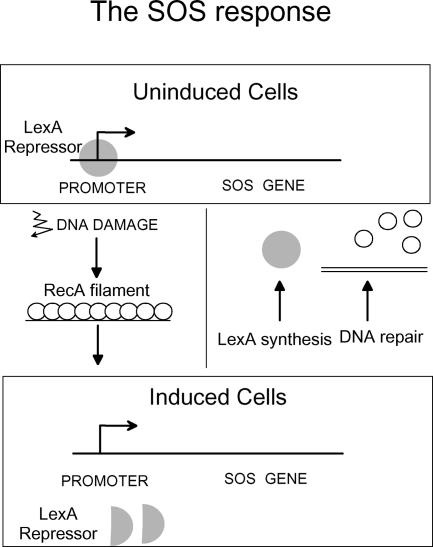
An Oscillatory Behavior for the SOS Response In non-induced growth conditions, the LexA repressor binds to SOS-controlled promoters, limiting or preventing their action. The basal level of expression of the genes that belong to the SOS regulon is variable. For example, in non-induced cells there are 7,500 molecules of RecA and undetectable amounts of Pol V. Upon DNA damage, RecA filaments formed at sites of damage activate the autocleavage of the LexA repressor, allowing SOS gene expression. SOS induction is reversed when damages are repaired. This is due to the disappearance of the RecA filament and allows the newly synthesized LexA molecules to bind SOS promoters. The recent work by Joel Stavans' laboratory provides evidence that, after DNA damage, individual cells oscillate between an induced and a less-induced state, and that the level of DNA damage governs the number of high-induced phases rather than their amplitude and timing [11]. Grey circles, LexA; white circles, RecA.

When the cell senses the presence of an increased level of DNA damage, the LexA repressor undergoes a self-cleavage reaction and the SOS genes are de-repressed (Figure 1). A nucleoprotein complex—the RecA filament—induces the LexA cleavage reaction. RecA is a ubiquitous protein, present in nearly all bacteria and conserved in all organisms, including humans. It specifically binds single-stranded DNA (ssDNA), forming a nucleoprotein filament that has two functions [2]: the RecA filament may either invade a homologous double-stranded DNA sequence and catalyze strand exchange (the key reaction of homologous recombination), or it may promote LexA cleavage (thereby inducing the SOS response). However, RecA binding to ssDNA is also regulated. It is prevented in vivo by the ubiquitous presence of the ssDNA binding protein. Two systems allow RecA to overcome the ssDNA binding protein barrier on certain substrates: the RecFOR proteins assist RecA binding to single-strand gaps, and the RecBC proteins directly load RecA on the processed double-strand ends. Consequently, DNA-damaging agents that induce the formation of DNA single-strand gaps, such as UV light, will induce the SOS response only if the RecFOR proteins are present, whereas those that create DNA double-strand ends, such as topoisomerase poisons, will require the RecBC proteins for SOS induction [3].

The SOS response has become a paradigm for the field of DNA repair. During the past 30 years, several laboratories have addressed questions concerning the function of the SOS genes and mechanisms that fine-tune their regulation. Classical techniques used to study the SOS response involved treatments of bacterial cultures by a DNA-damaging agent followed by analysis of reporter genes fused to an SOS promoter, or the direct quantification of LexA or RecA proteins by immunoblotting. More recently, microarrays were used to measure the timing and the amplitude of the induction in bacterial populations. About 40 genes were shown to be under SOS control. Most are DNA repair genes, but there are several genes that still have no known function [4,5].

## SOS Genes and Their Induction Order

After UV irradiation, the amount of LexA repressor decreases nearly 10-fold in a few minutes [6]. The SOS genes, however, are not all induced at the same time and to the same level. The first genes to be induced are *uvrA*, *uvrB*, and *uvrD*. These proteins, together with the endonuclease UvrC, catalyze nucleotide excision repair (NER), a reaction that excises the damaged nucleotides from double-stranded DNA. As a second defense against DNA lesions, expression of *recA* and other homologous recombination functions increase more slowly, about 10-fold. Homologous recombination allows the repair of lesions that occur on ssDNA regions at replication forks by rendering them double-stranded (and hence a substrate for NER). The division inhibitor SfiA is also induced to give the bacterium time to complete the repairs. Finally, about 40 minutes after DNA damage (and if the damage was not fully repaired by NER and homologous recombination), the mutagenic DNA repair polymerase Pol V (encoded from *umuC* and *umuD* genes) is induced [7]. This last-ditch response also allows bacteria to render DNA lesions double-stranded—hence reparable, but at the expense of introducing errors into the genome.

Clearly, it is important for bacteria to keep all levels of the SOS response under tight control; there is no utility to the organism of using error-prone polymerases longer than absolutely necessary. Therefore, it should come as no surprise that *lexA* itself is an SOS gene. The constant production of LexA during the SOS process ensures that as soon as DNA repair occurs, the disappearance of the inducing signal will allow LexA to re-accumulate and repress the SOS genes. Moreover, two SOS-induced proteins, DinI and RecX, affect the stability of the RecA filament and thus may participate in the control of the SOS response [8]. Finally, in addition to DNA-damaging agents, the inactivation of certain cellular functions causes chronic SOS induction. This may be either because the gene product is involved in DNA repair and in its absence spontaneous DNA lesions persist, or because the inactivated function is essential for proper DNA duplication and the replication defect increases the amount of ssDNA [9].

## Measuring the SOS Response

SOS induction was measured in bulk cultures until fluorescent microscopy techniques became available that allowed the direct measurement of gene expression in individual cells. Chronically induced cells were first used to measure SOS induction in single bacteria [10], where it was observed that the apparent homogeneity of SOS expression at the level of a population masked the occurrence of stochastic events in individuals. Indeed, the level of SOS expression in genetically identical cells grown in the same conditions was variable from cell to cell, with highly induced cells existing alongside non-induced ones. More recently, in this issue of *PLoS Biology*, Friedman and coworkers measured in single cells the level and kinetics of activation of SOS promoters after UV-light treatment [11]. To report promoter activity, the green fluorescent protein (GFP) gene was placed under the control of the promoters of three different SOS genes: *recA*, *lexA*, and *umuCD*. As expected, when the signal in a cell population was analyzed, the amount of GFP increased as a broad peak followed by a decrease as repair took place and the SOS response was shut off. Surprisingly, in individual cells, one, two, or three successive peaks of GFP expression were observed, depending on the UV dose. At UV doses lower than 10 joules (about 500 pyrimidine dimers per cell, where most should be removed by NER), one peak of GFP was observed. This was centered at 20 to 25 minutes after irradiation for the *recA* and the *lexA* promoter. Ten minutes later, as expected, the *umuCD* promoter was induced. At UV doses of 20 joules or higher, two to three peaks of GFP expression were observed, with the timing of the appearance of the first peak and its amplitude remaining constant. This finding changes our view of the control of the SOS response. It suggests that in each cell, the SOS response is not simply turned on to an extent that depends on the level of DNA damage and then turned off. Rather, it suggests that the SOS promoters are induced to a certain level sufficient to survive a certain dose of DNA-damaging agent, regardless of the initial amount of DNA damage. If the level of DNA damage is too high for the cells to cope with in one round of induction, a second round of induction or even a third round will follow. This interesting finding introduces a whole range of new questions, including: which factors are limiting the amplitude and controlling the timing of the peaks? The *umuC* and *umuD* genes seem to play a role in this process as their inactivation strongly perturbs the oscillatory behavior of the *recA* promoter. However, several models are possible as UmuC and UmuD act as a regulatory complex and as a lesion-bypass DNA polymerase [12]. Other SOS-induced proteins such as RecX and DinI that act on the RecA filament could be involved in this regulation [8]. Interestingly, digital oscillations were found also in human DNA repair governed by p53 [13], raising a parallel between the complex regulation of eukaryotic cells and the well-characterized, easily amendable SOS response of bacterial cells.

## SOS and Bacterial Resistance to Antibiotics

Beyond being a model of a DNA repair regulatory network, the SOS response has played an important role in shaping the bacterial world. This is mainly because it increases mutations and genetic exchanges [14]. Pol II, Pol IV (*dinB*), and Pol V (*umuCD*) are E. coli SOS-induced DNA polymerases that are able to replicate across lesions (bypass polymerases). Among them, only Pol II is induced early and has a high fidelity on intact DNA. In UV-treated cells, these DNA polymerases can induce mutations at the site of the lesion (targeted mutations) or elsewhere (untargeted mutations); their action may be coupled to the repair of DNA double-strand breaks by homologous recombination.

**Figure 2:**
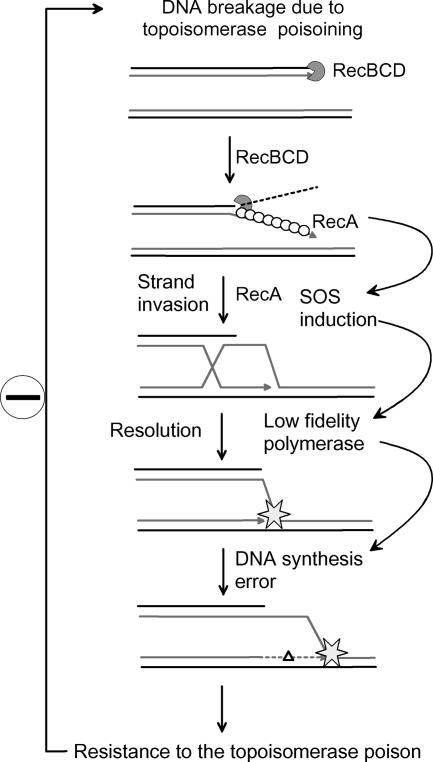
A Model for SOS-Dependent Evolution to Antibiotic Resistance Topoisomerase poisoning agents cause DNA double-strand breaks. RecBC in turn loads RecA. RecA filaments induce the SOS response and recombine. The homologous recombination reaction ends with a primer-template structure to which SOS-induced polymerases have access. DNA synthesis by these low-fidelity polymerases is accompanied by the introduction of mutations. A sub-population of mutant cells that can resist the poisoning agent invade the niche. The break-induced erroneous repair model, originally proposed for the mutagenic effects of DNA double-strand breaks in various laboratory conditions, accounts for the emergence of ciprofloxacin-resistant bacteria in a murine infection model [18]. Indented circles, RecBC; stars, SOS-induced DNA polymerases; triangle, mutation; white circles, RecA.

The repair of double-stranded DNA breaks necessarily involves a replication re-initiation step, which can be mutagenic (Figure 2). For example, when wild-type E. coli cells are placed in the presence of a carbon source that they cannot use, some of them suffer double-strand breaks in their chromosomes that are repaired by homologous recombination, creating a substrate for Pol IV. Due to the mutagenic action of this DNA polymerase, a sub-population of cells acquires the capacity to use the carbon source and propagates [15,16]. The mutator effect of Pol III mutations, which affect the main E. coli DNA polymerase and cause chronic SOS induction, also depends in part on the action of SOS-induced polymerases, even in the absence of external damage [17].

In the June issue of *PLoS Biology*, the Romesberg laboratory describes, using a murine infection model, a role for SOS induction in the appearance of E. coli mutants resistant to antibiotics [18]. The main antibiotic used is ciprofloxacin, a topoisomerase inhibitor that causes DNA double-strand breaks. The treatment of mice infections by ciprofloxacin leads to the rapid appearance of E. coli cells resistant to the antibiotic. Interestingly, when a pathogenic E. coli strain that encodes a non-cleavable LexA repressor is used, no ciprofloxacin-resistant mutant appears. This indicates that the formation of resistant cells requires SOS induction. A recombination- and SOS-dependent model is presented for the formation of ciprofloxacin-resistant mutants (Figure 2). Normally, topoisomerase poisoning causes the formation of DNA double-strand breaks, which in turn induce the SOS response and are repaired by homologous recombination. However, when the SOS response is triggered by antibiotic-induced DNA damage, the SOS-induced DNA polymerases that act at the replication forks formed by recombination generate mutants—some of which are resistant to ciprofloxacin. The SOS response is also involved, by other means, in the survival of E. coli in the presence of β-lactams [19,20]. These findings suggest that blocking SOS induction could be a general means to prevent the rapid evolution of bacteria to antibiotic resistance.

## Conclusions

Work on the SOS response illustrates well the dual purpose of bacterial studies, as SOS is both a modulator of bacterial propagation during pathogenicity, and an irreplaceable source of concepts for the understanding of DNA repair regulation networks. Several important issues remain to be addressed. For example, more than a dozen SOS-induced genes encode proteins of unknown function [4]. The identification of their physiological role may reveal new levels or new means of regulation of the SOS response, links with other cellular global regulation networks, and unsuspected consequences of the SOS induction.

## Abbreviations

GFP: green fluorescent protein
NER: nucleotide excision repair
ssDNA: single-stranded DNA.
